# Municipal Solid Waste Management through Sustainable Landfilling: In View of the Situation in Karachi, Pakistan

**DOI:** 10.3390/ijerph19020773

**Published:** 2022-01-11

**Authors:** Ihsanullah Sohoo, Marco Ritzkowski, Jinyang Guo, Kiran Sohoo, Kerstin Kuchta

**Affiliations:** 1 Circular Resource Engineering, Economy and Management (CREEM), Institute of Environmental Technology and Energy Economics, Hamburg University of Technology, Blohmstr. 15, 21079 Hamburg, Germany; m.ritzkowski@tuhh.de (M.R.); jy.guo@tuhh.de (J.G.); kiran.sohoo@hochschule-stralsund.de (K.S.); kuchta@tuhh.de (K.K.); 2 Department of Energy and Environment Engineering, Dawood University of Engineering and Technology, New M.A Jinnah Road, Karachi 74800, Pakistan; 3 School of Electrical Engineering and Computer Science, Renewable Energy and E-Mobility, Hochschule Stralsund—University of Applied Sciences, Zur Schwedenschanze 15, 18435 Stralsund, Germany

**Keywords:** open dump sites, sanitary landfills, bioreactor landfills, greenhouse gas emission, climate change, developing countries

## Abstract

Open disposal is the most common technique used for municipal solid waste (MSW) management due to the absence of sanitary landfills in Pakistan. The major cities and small towns in Pakistan have become a showcase of negligence and mismanagement of MSW, which results in deterioration of the environmental and social-life quality. Moreover, research has proved that inefficient handling (disposal) of MSW results in uncontrolled emissions of greenhouse gases (GHGs), mainly methane, and adds a significant share in global climate change. This study aims to estimate methane emissions from MSW disposed of at dumpsites and compare the GHG mitigation potential of different landfill strategies in specific climate and waste compositions in Karachi. The GHG estimations are based on lab-scale investigations conducted by simulating landfill conditions through the landfill simulation reactor (LSR) experiment. The synthetic MSW sample representing the composition of MSW generated in Karachi was used in the LSR experiment. Environmental sustainability and GHG mitigation potential of different landfilling strategies was evaluated by analyzing gas formation potential (GP_21_) and respiration activity (RI_4_) at the end of the experiment. This study revealed that the quantity of solid waste annually disposed of at dumpsites in Karachi possesses the potential to release about 3.9 Mt CO_2_-eq. methane (with specific methane potential of 1.8 tCO_2_-eq./tonne DM disposed) due to the biological decomposition of the organic fraction. Results show that the fresh waste disposed of at landfill sites in Karachi possesses about 92% and 94% higher GP_21_ and RI_4_, respectively, than the German allocated criteria for mechanically and biologically treated (MBT) waste for landfills Furthermore, sanitary landfills with post-aeration conditions showed higher GHG mitigation potential and low biological activity in the waste. The second highest GHG mitigation potential and lowest biological activity of the waste was noticed from bioreactor landfills with post-aeration conditions. The third number in GHG mitigation and reduced waste activity was noticed in the waste sampled from bioreactors without aftercare approach. The least GHG mitigation potential was noticed from the uncontrolled waste dumping (existing) approach with high residual gas potential and respiration index level. This lab-scale landfill simulation study can provide baseline data for further research and planning the development of new sustainable landfills in Karachi, Pakistan and in the region.

## 1. Introduction

Due to increasing population, rise in economic development, and rapid urbanization, resource consumption has intensified, and an increase in the global waste generation rate has been observed [1]. Hence, environmental issues associated with waste management are raising serious concerns [2]. Sustainable management of municipal solid waste (MSW) is one of the major challenges responsible authorities face in developing countries [3,4,5]. The primary goal of solid waste management is to deal with the environmental, public health, resource, aesthetic, land-use, and economic issues related to inadequate waste management practices [6]. Any single waste disposal approach cannot deal with waste materials in an environmentally sustainable way [7]. Therefore, an integrated waste management approach is widely recommended for sustainable waste management [8]. Integrated solid waste management can be described as selecting and applying suitable approaches and technologies to meet specific waste management objectives and goals through considering environmental and public health concerns [9]. However, adaptation of the waste management and disposal strategies vary by the economic level of the countries [10].

As a comparatively inexpensive technology for waste treatment and disposal, landfilling has been opted for globally, particularly in developing nations [11]. Hence, in global waste management strategies, about 37% of MSW is disposed of in landfills, and 33% of MSW ends up at open dumps [12]. Therefore, reducing landfill emissions is a fundamental goal in the waste management strategy that is also climate protective [13], as the waste management sector is contributing up to 5% of global emissions [14].

Like many developing countries, the waste management situation in Pakistan has also deteriorated due to political negligence, lack of finance and technology, public awareness and behavior, and administrative issues [15,16,17]. Open disposal is the most common technique for MSW management due to the absence of sanitary landfills in Pakistan [18,19]. The major cities and small towns in Pakistan have become a showcase of negligence and mismanagement of MSW generated, causing a significant impact on environmental and social-life quality.

According to the Ministry of Climate Change of Pakistan [20], the total GHG emissions in Pakistan for the year 2015 were 408.1 MtCO_2_-eq. In the total quantity of GHG emissions in Pakistan, the contribution of the waste sector is 15.5 MtCO_2_-eq. with methane (CH_4_) 13.4 MtCO_2_-eq. and nitrogen oxide (N_2_O) 2.1 MtCO_2_-eq, where disposal of MSW is causing 12.5 MtCO_2_-eq. of CH_4_ emissions [20]. Hence, in the international context of CH_4_ emissions from MSW handling and disposal, Pakistan is contributing 0.64% share in global CH_4_ emissions associated with waste handling and disposal.

A study by Korai et al. [21] reported that in Pakistan, 170 landfill sites are required to dispose of about 30.8 million tonnes of MSW generated annually throughout the country, achieving more than 90% of waste collection efficiency. Presently, Karachi does not have any adequately engineered landfill facility for municipal solid waste disposal, and all available waste landfilling sites are open dumps [19,22].

Reliable data about waste management are essential for a comprehensive, critical, and informative assessment of waste management options in every waste management programme [23]. These fundamental data about MSW management are lacking in Pakistan [24,25]. Nevertheless, some studies regarding waste management in Karachi have been published in recent years (2015–2020), in which mainly the status quo of MSW management in the city and energy generation from MSW were reported [24,26,27,28,29,30]. Furthermore, the waste management situation and challenges in Karachi were reported by [26,28,31].

However, in previous studies, GHG emissions from solid waste landfill (dump) sites in the existing situation (waste composition and climate) were not covered. Therefore, there is still a gap in comprehensive knowledge about the GHG emissions potential from waste disposed at landfill (dump) sites in Karachi. In this course, there is a pressing need for more comprehensive research to assess GHG emissions related to waste disposal and to propose strategies for their mitigation through the creation of new sustainable landfills for the city. This assessment can be done by comparison of different GHG mitigation approaches focusing on landfills. Hence, this research tackles the issue of missing data of GHG emissions from landfills in Karachi employing simulated landfills. The ultimate motivation behind this research is to propose an environmentally sustainable landfill strategy focusing on GHG emissions control and improved environmental behaviour.

Solid waste management through landfilling requires estimations of landfill gas, particularly methane emissions, to evaluate compliance with regulatory air quality standards [32]. However, the quantity of GHG emissions from waste disposed at landfill sites in Karachi is unknown. Therefore, to evaluate the environmental footprint of waste dumped, it is vital to know the amount of GHG emissions, especially methane released from waste disposal sites under prevailing conditions (climate and waste composition).

The aims and objectives of this study are to assess the GHG emissions potential of the waste disposal sites in view of the waste management (quantity and composition) and specific climate conditions in Karachi and compare different GHG mitigation approaches with a focus on landfills (anaerobic landfills and aerated landfills) for Karachi. This study can contribute to future research, planning, and design of new sustainable landfills in Karachi, Pakistan, and in the region.

## 2. MSW Management Situation and Climate Conditions in Karachi, Pakistan

Pakistan is considered a lower-middle-income country and is engulfed in severe environmental and public health problems due to the absence of sustainable waste management policy and infrastructure [19,22]. As a result, public concerns regarding uncontrolled and open waste disposal are growing with time [21]. The heaps of solid waste pilling up is commonly observed in major cities and small towns of the country like Karachi, Lahore, and Islamabad [33]. This study is conducted by considering the situation of Karachi, the most populous city and economic heart of the country, as a case study. The following sections provide insightful information regarding the geological location, climate conditions, and waste management situation of the city.

### 2.1. Geographical Location

**Figure 1:**
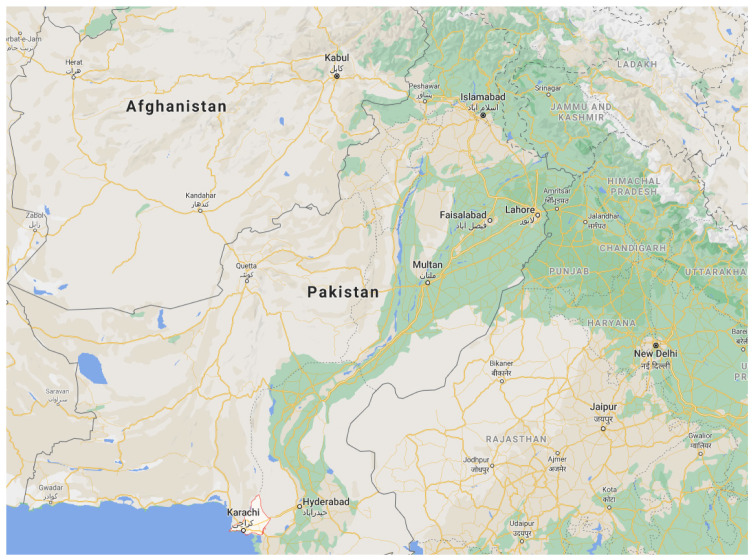
Location of Karachi city in the map of Pakistan (Google maps).

Karachi is the capital city of Sindh province in Pakistan. Karachi is the largest and most populated city of the country, located on the coast of the Arabian Sea as shown in Figure 1 [33]. According to KDA [34], Karachi city is spread over an area of 3530 square kilometers.

In the latest census of 2017, the population of Karachi was reported as 16.05 million, with an annual growth rate of 2.6% [35]. This increasing population places burdens on resources and waste management capacities.

### 2.2. Weather Conditions

**Figure 2:**
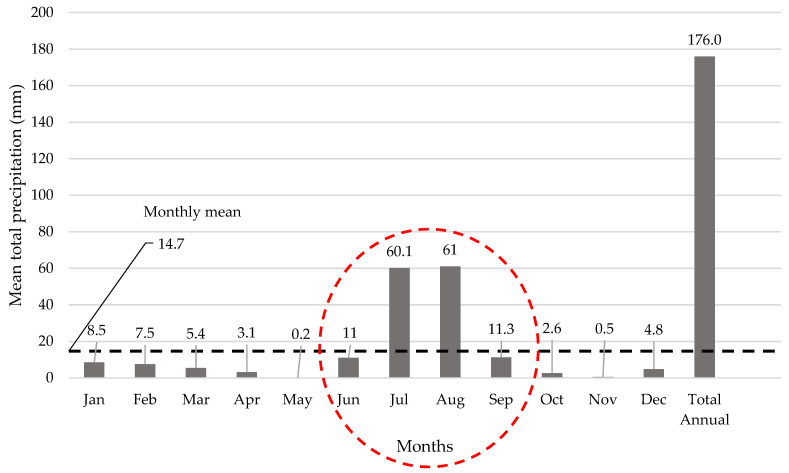
The average monthly rainfall in Karachi city–World Meteorological Organization (adapted from [37]).

Karachi city is located in the arid to hyper-arid climate region of Pakistan [36]. According to the World Meteorological Organization [37], the amount of annual mean rainfall in Karachi is 176 mm. Figure 2 shows the annual mean and monthly mean precipitation rate in Karachi city.

The monsoon period in Karachi typically occurs from June to September, when higher precipitation is recorded (as shown in Figure 2). The cumulative rainfall in the monsoon period is computed as 143.4 mm (with an average of 35.8 mm/month), when 81% of total annual rainfalls occur. As a result, during this period, higher leachate formation can be expected at waste disposal sites because presently no cover system (daily or final cover) is applied to control the liquid infiltration in the waste mass.

As discussed above, the major quantity of annual water budget of solid waste disposal sites in Karachi is contributed by precipitation in the monsoon season. Therefore, the estimations for theoretical water infiltration are made only for the peak precipitation period from June until September. Further assumptions, including the quantity of water coming by precipitation in the remaining months (from October until May) of the year, are balanced with evaporation and absorption by the solid waste. Hence, the theoretical water infiltration rate at waste disposal sites in Karachi is calculated as 1004 m^3^/ha (251 m^3^/ha/month) during 4 months of peak rainfall in the monsoon season (June–September).

**Figure 3:**
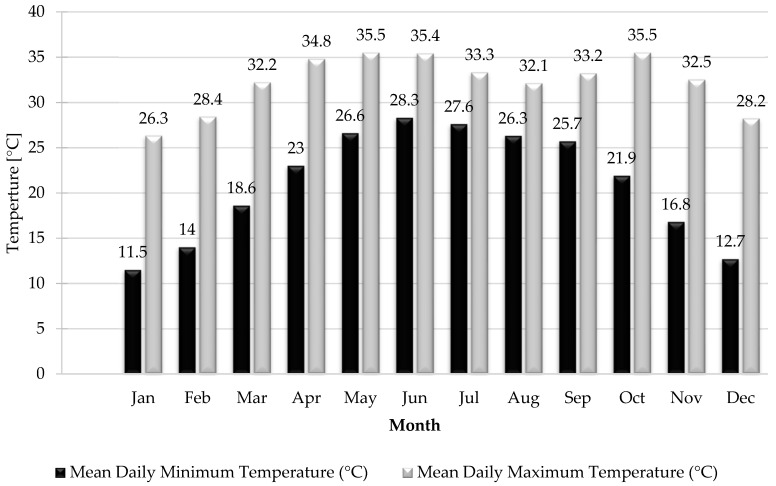
The mean daily temperature in Karachi–World Meteorological Organization (adapted from [37]).

The daily minimum mean temperature is 21.1 °C and the daily maximum mean temperature is 32.3 °C [37]. Figure 3 illustrates the graphical view of the daily mean minimum and maximum temperature in the Karachi city.

The transformation process of water into vapors due to solar radiation is referred to as evaporation and by vegetation (plants) as transpiration [38]. The combination of both phenomena is called evapotranspiration. According to Saifullah Khan and Mahmood Ul Hasan [39], the mean annual evapotranspiration rate in Karachi is 4.9 mm. The same study reported that the evapotranspiration in Karachi increases from 3.7 mm in February to 7 mm in May and then decreases until 3.2 mm in January [39].

Furthermore, authors [39] reported the average evapotranspiration rate in Karachi city is 3.8 mm in winter and 5.7 mm in summer. The key factors influencing the evapotranspiration rate are temperature, rainfall, sunshine duration, relative humidity, surface pressure, wind speed, fog, cloudiness, topography, and latitudinal and longitudinal degrees [39].

### 2.3. MSW Management and Treatment

Several studies [40,41,42,43,44] confirmed that Karachi is facing severe environmental challenges. Overflowing sewers, rain canals blocked with trash, streets and roadsides with a view of waste dumpsites, and air pollution are the most environmentally devastating problems in the city.

Sustainable management of a huge amount of MSW generated daily has become a challenging task for the municipal authorities of Karachi. Most municipal authorities do not maintain records of the waste quantity, composition, and characteristics. The absence of basic information is one of the major hindrances in properly planning for waste treatment and disposal facilities [31]. Sindh Solid Waste Management Board (SSWMB) was founded as an independent agency in 2014. It is responsible for managing solid waste in the Sindh province [45].

According to SSWMB [45], MSW generated in Karachi is managed by three operational stages, named as front end services, middle end services, and back-end services. SSWMB has an institutional mandate for solid waste collection, transfer and transport, disposal, the operation of transfer stations, and the operation of landfill sites [45]. The front end services involve the collection of solid waste from the primary collection bins (door to door collection) and transferring it to designated garbage transfer stations (GTS). The middle end services are the operation of GTSs and transportation of solid waste from GTSs to landfills (waste disposal) sites for the final disposal. Finally, the back end services include operation and maintenance of waste disposal sites.

#### 2.3.1. MSW Generation

Compared to developed countries, the MSW generation rate in Pakistan is much lower [19]. However, due to being the most populous city and industrial hub of the country, Karachi is the largest MSW producing city in Pakistan. The MSW generation rate in Karachi is reported as 0.76 kg/capita/day [46]. Hence, overall 15,600 tonnes of MSW is being generated daily in the city [24].

#### 2.3.2. MSW Composition

A study [31] reported the average composition of MSW in Karachi as 51% of bio-degradable fractions and 49% of non-biodegradable fractions on a wet weight basis. The biodegradable portion of the MSW contains food waste, green waste, paper, and paper products. The non-biodegradable part comprises glass, metals, plastics, textiles, nappies, tetra packs, fines (stones, sand, and ashes), and wood waste.

#### 2.3.3. MSW Collection and Transfer

MSW collected from the community bins is transferred to six garbage transfer stations (GTS) located in each city district. Segregation of the MSW at source is not applied [45]. According to the report by [47], Sindh Solid Waste Management Board (SSWBM) is collecting about 8000–10,000 tonnes of solid waste from the primary collection points to the GTSs through private companies.

According to SSWMB [45], about 2000–2500 tonnes/day of solid waste is collected and disposed of by Karachi Metropolitan Corporation (KMC) and District Municipal Corporations (DMCs), and 1000–2000 tonnes/day of solid waste is collected and disposed of by the cantonment Boards in the city. Overall, 75% of the MSW generated in the city is collected [24]. Streets, roadsides, and vacant plots within the city areas are littered with the remaining uncollected waste [26,31]. Hence, the remaining uncollected waste is associated with various socio-environmental problems in the city [21,48].

#### 2.3.4. MSW Recovery and Recycling

In the absence of proper governmental policy and systems for waste recovery and recycling, only 26% (4100 tonnes/day) of the total amount of MSW generated in Karachi is recovered for recycling by informal waste sector [24]. As in many other developing countries, the informal sector in Pakistan is the key contributor to material recovery and is sometimes the only actor [14]. According to SSWMB [45], approximately 50,000 waste pickers operate in Karachi and each of them is collecting about 60–100 kg of recyclables on a daily basis.

Mostly, the recyclable material, especially metals, paper, and plastics, are separated at source or by waste pickers (at community bins) and then sold to junk shops locally called “Kabari” [19,28]. At this initial stage of the waste management system, the volume of waste is reduced due to primary separation [28]. At the later stage, uncollected recyclable materials remaining in the solid waste is picked by scavengers at landfill sites [24]. 

Overall, 83.5% (3,422 tonnes/day) of the total material recovered from the MSW is informally recycled. The remaining 16.5% (678 tonnes/day) of recovered material is rejected due to process limitations/inefficiencies and is finally disposed of in landfills again [24].

#### 2.3.5. MSW Disposal

In the existing waste management system, which is controlled by SSWMB, the solid waste received at garbage transfer stations is transported to designated official MSW landfill sites [45]. As Karachi city has no well-designed solid waste collection and disposal system [19,49], all collected solid waste is openly disposed of in landfills (dump sites) without any cover.

Karachi has two official landfill sites (500 acres each) for the disposal of solid waste generated in the city—operated and maintained by SSWMB. One is Jam Chakro in Surjani town, and the other is Gond Pass along the Hub river road (Northern Bypass) [45]. However, the waste of Karachi is being dumped at another 10–20 unofficial waste disposal sites in and around the city [26,50]. The major unofficial waste dumpsites in Karachi are Ibrahim Hydri, Rehri Goth [51], Mehmoodabad, Orangi, Meva Shah [26], Korangi and Lalabad Landhi [52]. All waste generated from different sources, including construction, industrial, and hospital waste, are openly dumped in landfills without any separation or compaction [31].

According to SSWMB [45], landfill sites Jam Chakro and Gond pass landfills are receiving 8000 tonnes/day and 3000 tonnes/day, respectively. An official from SSWMB stated in a telephone interview that both official solid waste disposal facilities (Jam Chakro and Gond Pass) have been operated as controlled landfill sites since the year 2017. The control measures adopted by SSWMB includes recording the quantity of solid waste by means of a weight bridge, spreading of the disposed waste, compacting (value is unknown), weekly soil cover (6 inches), fire monitoring and control, and placing a responsible person of SSWMB on 24 h on duty at a control room for site monitoring.

## 3. Materials and Methods

### 3.1. MSW Sample Modelling

**Figure 4:**
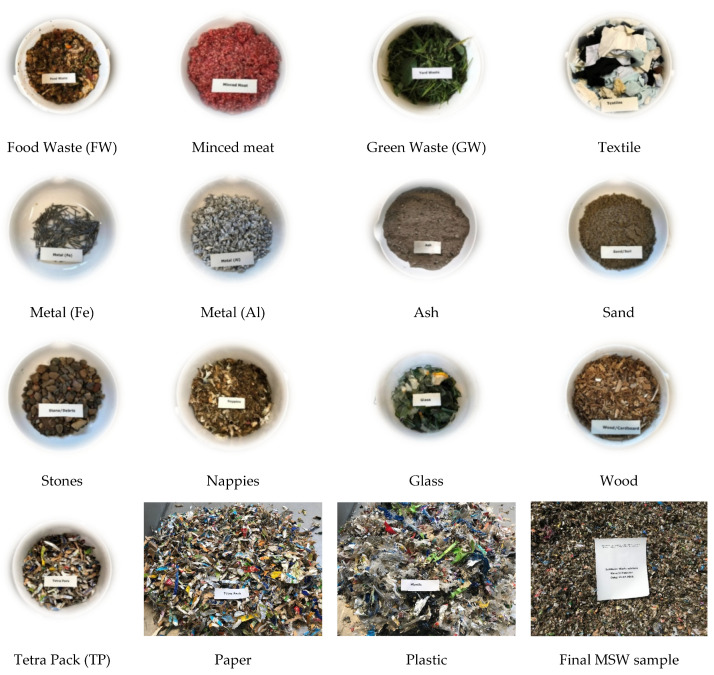
Components of MSW waste sample used in landfill simulation experiment—adapted with permission from Ref. [33]. Copyright 2020 Elsevier Ltd.

Various studies [53,54,55,56] used synthetic waste samples to conduct landfill simulation reactor investigations. The synthetic waste samples represented the typical composition of MSW generated in the study location [55]. This study also used a synthetic waste sample, which was prepared in the laboratory according to the MSW composition (% wet weight basis) in Karachi reported by [31]. The composition of the synthetic waste sample used in this study is given in Table 1. For modeling of the synthetic waste, samples of different waste components (shown in Figure 4) were utilized to prepare a 30 kg representative MSW sample as waste composition in Karachi city [57]. Further details about modeling of synthetic waste sample is reported in another research article [33].

Before mixing, the size of individual waste material was reduced either by manual cutting or by shredding with a shredder to approximately ≤25 mm particle size, and the waste sample was collected to investigate initial moisture content [57]. To increase the specific weight and moisture content of the waste as reported in [58], ten litters of tap water were then added to the prepared waste sample, and waste was thoroughly mixed to ensure equal distribution of moisture in the waste [57]. After moisturizing and homogenizing the prepared waste mixture, the waste sample was collected again for physicochemical analysis of the synthetic waste [57]. Physicochemical characteristics of modeled waste samples used in landfill simulation reactors are presented in Table 2.

The weight of all reactors was measured before and after loading the waste sample with an electronic balance. The prepared waste material was filled in the reactors manually and was slightly compacted through moderate pressing with a wooden stick [57]. The waste height in the reactors was noted after loading to calculate the waste volume and density. Basic details of waste loaded in landfill simulation reactors are given in Table 3. After loading the waste in the reactors, the field capacity of the waste was adjusted by adding 1 L of tap water in each reactor in correspondence with Ritzkowski et al. [60]. Subsequently, a 250 mL sample from drained leachate was collected for initial analysis [57].

### 3.2. Landfill Simulation Experiment Methodology

**Figure 5:**
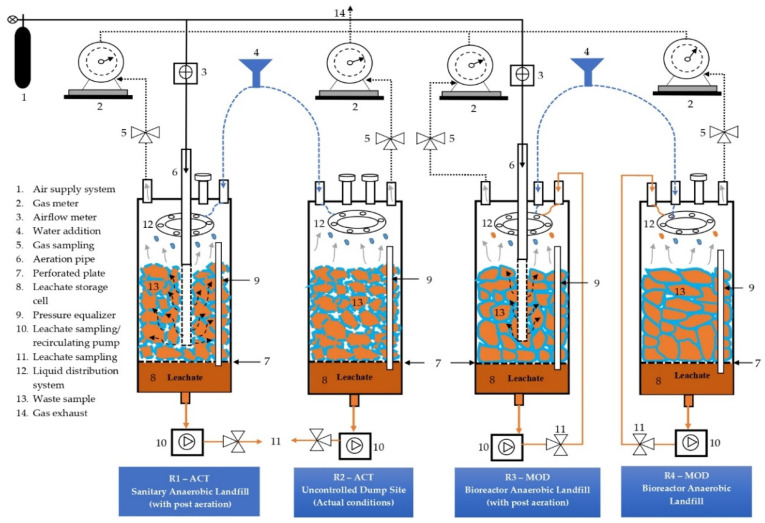
Schematic of experimental setup of landfill simulation reactors (LSRs)—adapted with permission from Ref. [59]. Copyright 2021 Elsevier B.V.

In this research, four different conditions were simulated in lab-scale reactors representing the open dumpsites, sanitary landfill with post-aeration, anaerobic bioreactor landfill, and bioreactor landfill with post-aeration to investigate the GHG emissions behavior and stabilization level of MSW under each condition. In a landfill simulated reactor named R2-ACT, uncontrolled dumpsite conditions were simulated to investigate the potential of GHG emissions from waste disposal sites in prevailing conditions (climate and waste composition) in Karachi, as illustrated in Figure 5. Landfill simulation reactors (LSR) simulated three types of landfills to assess the GHG mitigation potential of landfills under the situation in Karachi. For each case, one reactor was used. The reactor R1-ACT represented a sanitary anaerobic landfill with aftercare (post-aeration), reactor R3-MOD represented a bioreactor landfill with aftercare (post-aeration), and reactor R4-MOD represented an aerobic bioreactor landfill without aftercare.

The landfill simulation (LSR) experiment was conducted in a room with controlled climatic conditions at the temperature in the mesophilic range of 36 ± 1 °C [57].

#### 3.2.1. Pre-Aeration Operation

As the MSW sample used in this study was fresh and contained a high organic fraction, a short pre-aeration operation phase was realized in all the landfill simulation reactors to simulate initial aerobic decomposition of solid waste at open dumpsites, avoid unnecessary long lag phases, and reduce the intensity of acid formation anaerobic conditions [57,59,61]. In addition, various studies [61,62] suggested the pre-aeration operation as a pre-treatment of fresh waste (primarily comprising high organic content) prior to the anaerobic phase of landfill operation.

Furthermore, Ref. [63] reported that the pre-aeration phase aims to decrease the volatile fatty acids (VFA) level and increase the pH level, consequently promoting methane generation in the anaerobic phase. As described previously, pre-aeration is an effective approach for controlling the degradation of municipal solid waste with high organic fraction in the successive anaerobic phase of landfill operations [64]. At the commencement of the pre-aeration operation, 500 mL tap supplement water was added to each reactor through the liquid distribution system to obtain additional liquid for leachate recirculation [57]. The operation details of the pre-aeration phase are summarized in Table 4.

#### 3.2.2. Anaerobic Operation

Following the pre-aeration phase, the anaerobic phase was started by flushing all reactors with nitrogen (N_2_) gas for 15 min to purge oxygen and establish complete anaerobic conditions in the reactors [57,65]. In the first two reactors (R1-ACT and R2-ACT), 56 mL/week tap water was added to simulate the annual rainfall in the local situation (176 mm/a) [37,57]. In the other two reactors (R3-MOD and R4-MOD), 165 mL tap water was added weekly to provide process water for the recirculation system to observe the enhanced leaching effect, as a common practice in landfill simulation experiments [66].

The enhanced leaching facility aims at establishing optimal conditions for biodegradation and leaching of dissolved compounds [67]. Recirculation of leachate was carried out two times per day (12:00 and 24:00) in R3-MOD and R4-MOD reactors. For the complete analysis set, 250 mL of leachate was sampled once a month from all the reactors and preserved in 250 mL plastic bottles at 4 °C [59]. In addition, the quantity and quality of off-gas from all reactors were measured once a week through mini-gas counters and gas chromatography (HP-5890), Agilent, respectively [57].

#### 3.2.3. Post-Aeration

The post aeration phase was started in reactors R1-ACT and R3-MOD after 252 days of anaerobic operation when the weekly biogas production rate from these two reactors reached less than 0.5% of the cumulative biogas produced during the anaerobic phase [59]. The post-aeration phase aimed to observe the effects of aeration on landfill emissions and waste stabilization in sanitary landfill and bioreactor landfill conditions [59].

The aeration rate in the reactors R1-ACT and R3-MOD was 8.28 L/kg DM/day and 6.87 L/kg DM/day, respectively [59]. The volume of off-gas from aerated landfill reactors was measured using drum gas meters [57,65]. The frequency of off-gas analysis and measurement was the same as followed in the anaerobic phase. The operation of landfill simulation experiment operation is summarized in Table 5.

#### 3.2.4. Completion of the Landfill Simulation Reactor Experiment

After 364 days, the experiment of LSR simulating bioreactor landfill conditions in R3-MOD and R4-MOD was completed. The operation of the remaining two reactors (R1-ACT and R2-ACT) ended after 448 days. At the end of the experiment, reactors were opened, and waste height in each reactor was recorded. After that, each reactor was weighed individually to determine the losses in waste mass during the experimental phase.

Immediately after the opening, the reactor waste sample was collected to analyse total solids (TS) and volatile solids (VS). The remaining waste samples were placed in airtight plastic bags and stored at 4 °C for the final analysis of total organic carbon (TOC), gas formation potential (GP_21_), and respiration activity (RI_4_ and RI_7_).

### 3.3. Assessment of GHG Emission Potential and Biostabilization of MSW

The GHG mitigation potential of each landfill approach investigated in this research was compared based on results obtained by conducting a residual gas potential analysis (GP_21_) test of solid waste after the landfill simulation reactor (LSR) experiment. The residual gas potential analysis (GP_21_) results were compared with the target value of (GP_21_) proposed in the literature. Furthermore, the extent of bio-stabilization of the solid waste extracted from each LSR was experimentally investigated by conducting a respiration activity test (RI_7_). Results were compared with limit values suggested for the completion criteria of active landfill in-situ reported in the literature.

#### 3.3.1. Gas Formation Potential Assessment (GP_21_)

The 21 days biogas formation potential (GP_21_) test, also known as biochemical methane potential (BMP) test, is a rapid, economical, and established laboratory test method to assess anaerobic biodegradability of solid waste [68,69,70]. Furthermore, researchers including Angelidaki et al. [71] and Labatut et al. [72] described the BMP test as a short-term (i.e., 1–2 months) batch type anaerobic test to assess the methane generation potential and bio-stabilization of substrates. Another study [69] used the gas formation test to compare landfill performance, where BMP assessment was used to determine the initial and residual methane potential of the solid waste sample during 27 months of a pilot-scale LSR experiment.

Similarly, in this study, the initial biogas (within 21 days—GP_21_) formation potential of a fresh waste sample and residual gas potential of digested waste material obtained from different landfill simulation reactors was determined to compare the residual gas potential of waste samples. The GP_21_ test of fresh and digested waste samples was conducted following VDI 3640 protocol [73]. The volume of gas produced under standard conditions is determined according to DIN 38 414–8 using Equation (1).

(1) $$V_{tr,N}=V\times \left(P-Pw\right)\times \frac{T_{N}}{P_{N}}\times T$$

where *V_tr,N_* is the volume of dry gas in normal conditions (mL_N_); *V* is the read of volume of gas (mL); *P* is the air pressure at the time of reading (hPa); *Pw* is the vapour pressure of water at ambient temperature (hPa); *T_N_* is the standard temperature, (*T_N_* = 273.15); *P_N_* is the standard pressure, (*P_N_* = 1013 hPa); and *T* is the temperature of the climate room at the time of reading (K). The specific gas production (*V_S_*) from anaerobic digestion was determined by using Equation (2).

(2) $$V_{S}=\sum^{\;}V_{n}\times \frac{10^{4}}{m}\times TR$$

where *V_s_* is the specific gas formation relative to dry mass during the test period (mL_N_/g DM); ∑*V_n_* is the sum of the net volume of gas produced during test period (mL_N_); *m* is mass of the sample used (g); and *TR* is the dry mass in sample (%). The net gas volume produced from the normal conditions is determined as the difference between the normal volume of gas from substrate and the normal volume of blank (sludge) during the test period.

#### 3.3.2. Respiration Activity (RI_4_ and RI_7_)

The respiration activity of waste material is investigated to describe its biological stability [74]. This respiration index (RI) test was conducted with a batch test using pressure sensors from Oxytop system (WTW, Germany) under relevant German regulations DIN 29408 [75]. This aerobic test is employed to determine waste behavior under landfill conditions in a short period using standardized methods [74]. Furthermore, this test method also assesses the biological activity from the mechanically biological treated (MBT) waste materials [74]. This respiration test estimates the amount of residual organic material in waste to be potentially degraded under aerobic conditions. The respiration index test of the prepared sample was conducted to determine oxygen consumption potential and biological activity of the fresh waste material used in this experiment. The respiration activity test was conducted for four and seven days in constant temperature of 20 ± 1 °C. Equations (3) and (4) are used to calculate the oxygen consumption.

(3) $$AT_{4}=\frac{M_{R}\left(O_{2}\right)}{R\times T}\times \frac{V_{fr}}{m_{DS}}\times ∆p$$

where *AT*_4_ is the respiration activity of waste sample after 4 days (mg O_2_/g DM]), *M_R_*(O_2_) is the molar mass of the oxygen, 32,000 (mg/mol); *R* is general gas constant (83.144 L × hPa/mol × K); and *T* is the temperature of incubation (293.15 K);

(4) $$AT_{4}=K_{20{}^{\circ}C}\times \frac{V_{fr}}{m_{DS}}\times ∆p$$

where *K*_20°C_ is = *M_R_*(O_2_)/(*R* × *T*); absorbent and absorbent medium; *V_fr_* is the free gas volume (L); *m_DS_* is the dry mass of waste sample (g); and ∆*p* is the pressure decrease in test glass without waste sample.

The biogas composition (N_2_, O_2_, CO_2_, CH_4_) during the landfill simulation experiment and GP_21_ assay was analyzed by gas chromatography (HP-5890), Agilent. Total solids (TS) and volatile solids (VS) were analysed according to protocols DIN 38 414–S 2 and DIN 3809–H 1–3, respectively [60]. Total carbon (TC) and total inorganic carbon (TIC) were investigated by DIN EN 15936 with Multi EA 4000 Analyser [59,60]. The calorific value of the waste material was analysed in accordance with DIN EN 51900 employing IKA C 5000 (IKA-Werke GMBH&CO.KG, Baden-Württemberg, Germany) [60].

## 4. Results and Discussion

### 4.1. Production and Composition of Landfill Gas during Anaerobic Operations

The landfill gas (LFG) production rate from reactors R3-MOD and R4-MOD (with leachate recirculation) was significantly higher than for reactors R1-ACT and R2-ACT at the beginning of anaerobic operations. The rate of LFG production sharply increased in R3-MOD and R4-MOD and reached the maximum values of about 0.18 and 0.16 L/kg DM/h, respectively, during the initial 28 days of anaerobic operations.

**Figure 6:**
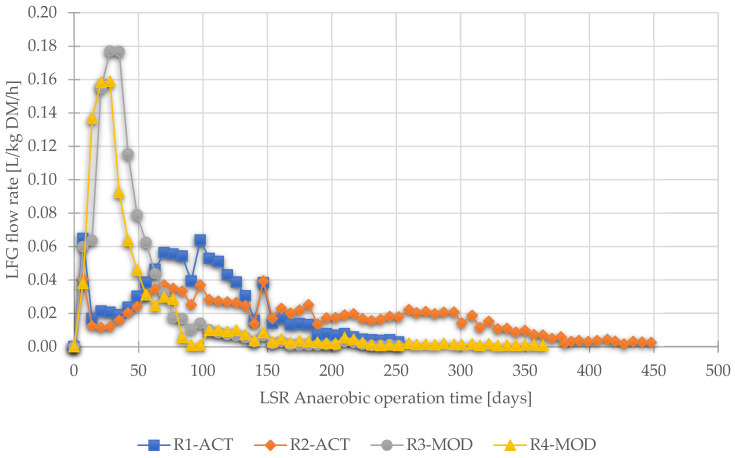
Evolution of landfill gas flow rate during anaerobic operation.

Afterward, the gas production showed a continuous decline, and the weekly gas production rate declined to <0.5% of cumulative gas production in 133 days. According to another study [76], this rapid decline in biogas production shows quick depletion of organic carbon due to the flushing effect. In contrast, an initial decline was noticed in biogas production rate in reactors R1-ACT and R2-ACT during the initial 28 days as shown in Figure 6. Subsequently, gas production was gradually increased in the reactors and reached maximum values only of 0.06 and 0.04 L/kg DM/h in R1-ACT and R2-ACT, respectively. The level of landfill gas production < 0.5% of cumulative gas production in reactors R1-ACT and R2-ACT took, on average, 26% more time to reach (189 and 373 days, respectively) in contrast to R3-MOD and R4-MOD.

The leachate recirculation and excess water addition in reactors R3-MOD and R4-mod accelerated the biological processes, resulting in higher biogas production. According to [67], a more conducive environment can be provided to the microorganisms in landfill by leachate recycling and adding excess moisture.

**Figure 7:**
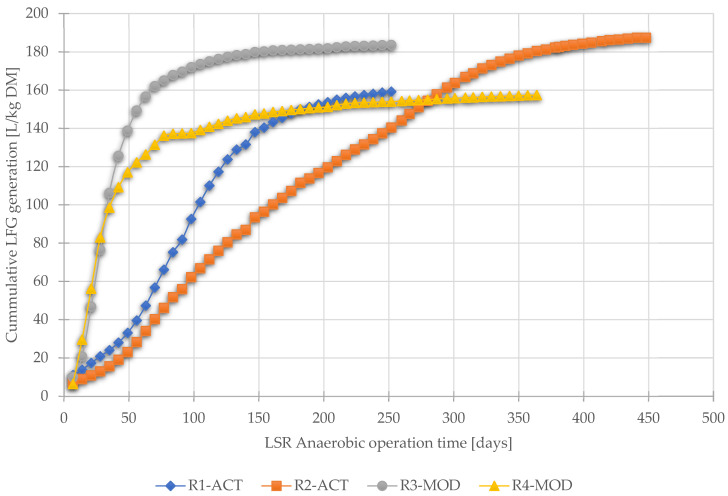
Cumulative landfill gas generated during anaerobic operation, updated (adapted from [57]).

However, as shown in Figure 7, this initial lag (for 28 days) in gas production from reactors R1-ACT and R2-ACT (operated as traditional landfills) was noticed due to hydrolysis and formation of organic acids [54]. In the anaerobic decomposition phase, the cumulative landfill produced from the reactors was recorded as follows: R1-ACT 159 L/kg DM, R2-ACT 187 L/kg DM, R3-MOD 184 L/kg DM, and R4-MOD 157 L/kg DM.

The waste degradation phases can also be differentiated with landfill gas production and the fraction of CO_2_ and CH_4_ in biogas [77]. The methanogenic phase in landfill production is defined by a methane concentration of approximately 50–60% and carbon dioxide approximately 40–50% in biogas [78]. According to the results obtained, it was estimated that the methanogenesis phase in reactors R3-MOD and R4-MOD was reached after 20 days of anaerobic degradation, whereas the methanogenesis phase in R1-ACT and R2-ACT was reached in 50 days. Figure 7 shows the graphical view of cumulative landfill gas produced from different reactors during anaerobic operation.

The average composition (*v*/*v*) of CH_4_ and CO_2_ in landfill gas produced during anaerobic operation in R1-ACT was noted as 56.2% CH_4_ and 43.8% CO_2_, in R2-ACT 57.1% CH_4_ and 42.9% CO_2_, in R3-MOD 64.2% CH_4_ and 35.8% CO_2_, and in R4-MOD 67.6% CH_4_ and 32.4% CO_2_. Moreover, from analysis of these results, it is possible to determine that reactors equipped with a leachate recirculation facility (R3-MOD and R4-MOD) reached the methanogenesis phase in 60% less time than the reactors operated without this facility (R1-ACT and R2-ACT).

**Figure 8:**
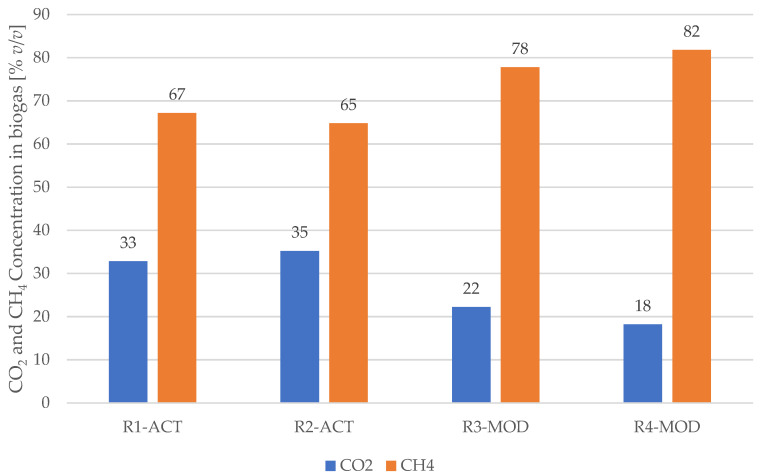
CO_2_ and CH_4_ concentrations in landfill gas at the end of anaerobic phase.

Similar outcomes are reported [77] where the methanogenesis phase was reached in 60% less time in reactors due to leachate recirculation. At the end of the anaerobic operation, CH_4_ and CO_2_ concentrations noted in LSRs are shown in Figure 8. The average CH_4_ concentration in the gas produced from LSRs simulating bioreactor situation was 23% higher (with 80% CH_4_ concentration) than LSRs simulating conventional/open landfill conditions where CH_4_ concentration achieved up to 66% at the end of the anaerobic phase.

Analysis of results obtained (LFG production and CH_4_ fraction of reactor R2-ACT) from this study and waste (moisture content and quantity) disposed at official dumpsites in Karachi revealed that waste dumpsites are potentially emitting 3.9 MtCO_2_-eq. methane annually. Table 6 presents the detailed calculations for greenhouse gas emissions estimated for the waste disposed of annually at dumpsites in Karachi.

### 4.2. Gas Formation Potential Assessment (GP_21_)

**Figure 9:**
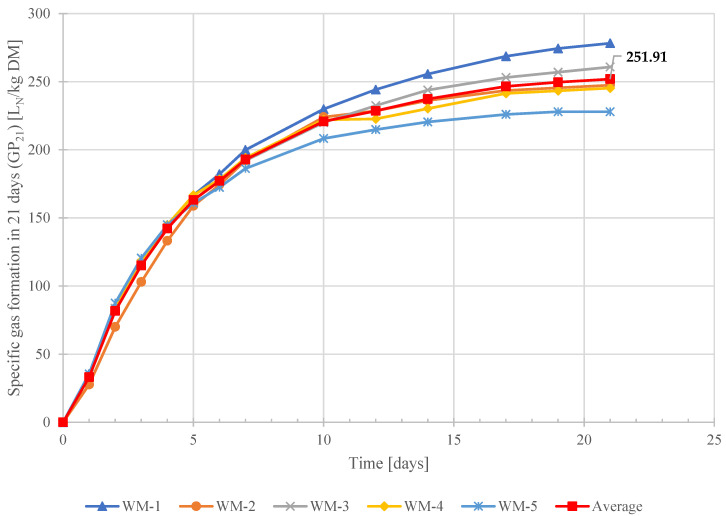
Net Specific gas formation from five replications of fresh MSW samples.

The average value of biogas formation potential from the fresh waste sample utilized in this study was 252 L_N_/kg DM. The graphs of net specific gas formation from five replicated fresh waste materials (WM) are illustrated in Figure 9. As regulation for stabilization criteria for municipal solid waste (e.g., GP_21_) is not available in Pakistan, the test results were compared with proposed limits of waste stabilization reported in the literature (according to the German regulation). In the German regulation for landfilling of pre-treated waste material, the proposed target value for residual gas formation potential (GP_21_) is ≤20 L_N_/kg DM for waste acceptance in landfills for final disposal [58]. However, an assessment is based on the GP_21_ value achieved from fresh waste material (WM); the waste dumped in landfill sites in Karachi has about 92% higher emissions potential than the limit value prescribed in German regulation.

**Figure 10:**
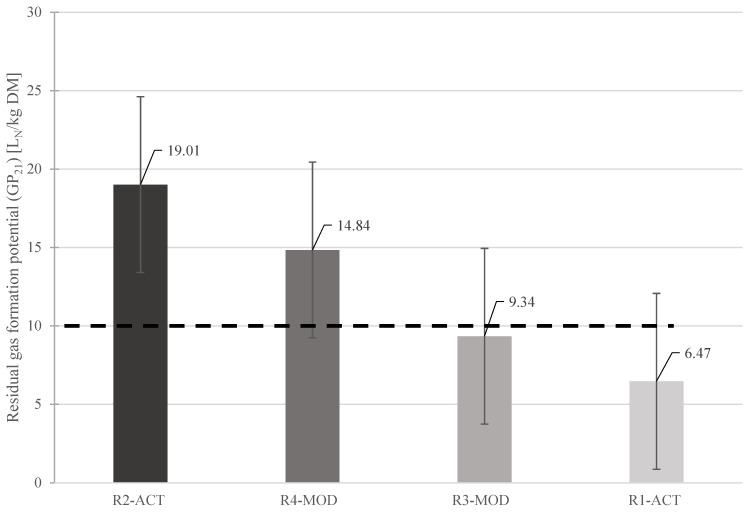
Residual gas formation potential of waste samples after experiment—adapted with permission from Ref. [59]. Copyright 2021 Elsevier B.V.

After completion of the experiment, the residual gas potential (GP_21_) value noted from the waste material sampled from reactor R1-ACT was well below the landfill aeration completion criteria of ≤10 L_N_/kg DM proposed by authors [80] (as shown in Figure 10) due to active post-aeration operation in the reactor. The residual gas formation from this reactor was reduced to 97.5% from the initial value of gas formation potential from the fresh waste sample loaded in the reactor [59]. This significant decrease in gas formation shows that the organic substance available for the anaerobic digestion process had already degraded to a large extent [58].

The waste sampled from reactor R3-MOD barely met the target limit for residual gas potential GP_21_ with 9.34 L_N_/kg DM [59]. In comparison, the waste samples from reactors operated under completely anaerobic conditions throughout the test duration (without post aeration operation) showed a higher residual gas potential from the limit value during the GP_21_ test. The waste sampled from reactor R2-ACT produced 19.01 L_N_/kg DM, and waste sampled from reactor R4-MOD produced 14.84 L_N_/kg DM [59]. Figure 10 shows the residual gas potential from waste sampled from different landfills regarding suggested GP_21_ criteria for waste stabilization.

### 4.3. Respiration Activity (RI_4_ and RI_7_)

**Figure 11:**
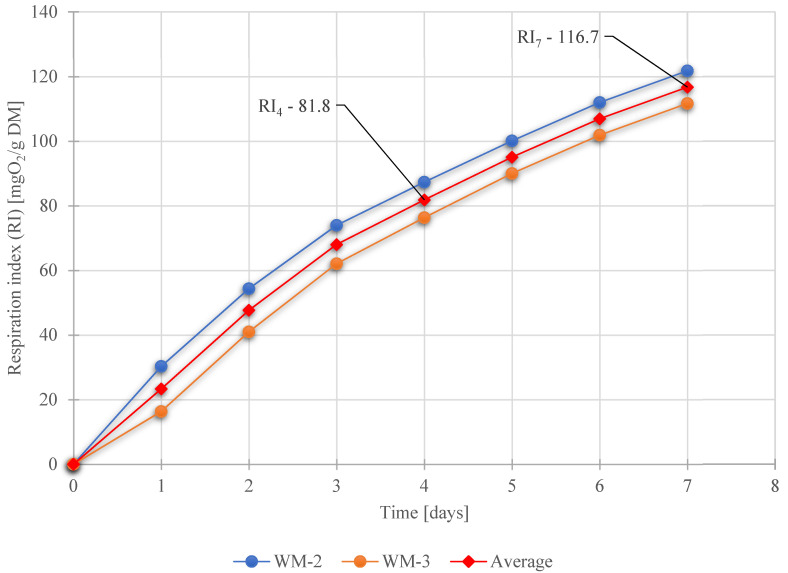
Respiration index (RI_4_ and RI_7_) of fresh waste samples.

The initial respiration index of fresh waste material (WM) for four days (RI_4_) was determined as 81.8 mgO_2_/g DM and for seven days (RI_7_) was 116.7 mgO_2_/g DM. The evolution in respiration activity of fresh waste material is shown in Figure 11.

It is assumed from the respiration index value achieved from fresh waste material that the level of respiration index in waste is being disposed of at waste disposal sites in Karachi is 94% higher than the reference limit value of ≤5 mgO_2_/g DM given for waste acceptance in landfills in the German regulation for landfilling of pre-treated waste material [58,63].

After the LSRs experiment was completed, the respiration activity of waste sampled from each reactor was determined in order to assess the biological stability of the waste degraded under different landfilling approaches. The seven day respiration index (RI_7_) decreased significantly from the initial level in all LSRs. However, the biological activity in waste sampled from R1-ACT was lower than waste sampled from all other reactors.

The value of respiration index (RI_7_) achieved in R1-ACT was ≤2.5 mgO_2_/g DM, which is in line with proposed value for ending of active in-situ aeration of waste in landfills reported in literature [80]. The test results show that a significant amount of residual organic material was degraded in the waste material sample from R1-ACT. Furthermore, it is assumed that up to 90% reduction in biodegradable organic carbon (BOC) was achieved in the waste material at the end of the experiment (post-aeration operation) in the reactor [80].

The respiration index (RI_7_) level of waste sampled from R3-MOD was higher than the limit value for the completion of landfill aeration with a value of 4.5 mgO_2_/g DM; even the post aeration phase was realized in the reactor. This phenomenon proves that the conditions in R3-MOD were not favorable for aerobic degradation (due to active aeration) of waste in the reactor. The one the factors involved in this phenomenon would be presence of significant moisture in waste mass as the reactor R3-MOD was simulating bioreactor landfill conditions with excess water addition and leachate recirculation.

It is evident from the result of respiration activity investigations that the supplied air in the reactor was not well distributed, and the assimilation of the air was limited by water coating the waste material [59]. As a result of limited air distribution, residual organics in the waste material were not sufficiently oxidized, and targeted waste stabilization was not achieved [59]. Therefore, the air distribution and assimilation should be optimized by draining out the supplementary water available in landfills prior to the start of in-situ aeration operation [59].

The respiration activity in both reactors R2-ACT and R4-MOD was higher than landfill stabilization criteria, with RI_7_ value of 5.3 mgO_2_/g DM and 4.8 mgO_2_/g DM, respectively. Both reactors were operated under anaerobic conditions throughout the experiment operation time. However, the lower value of RI_7_ in the waste sampled from the reactor R4-MOD than the value in the waste sampled from the reactor R2-ACT is a result of the operation conditions in the reactors.

**Figure 12:**
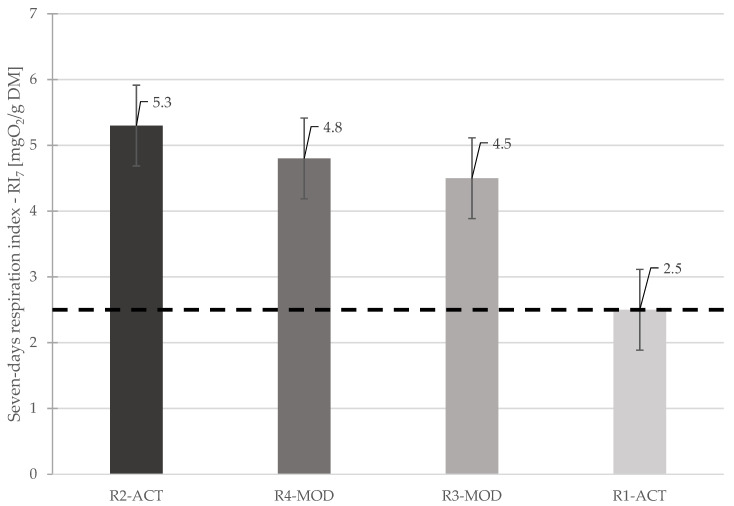
Comparison of seven day respiration index (RI_7_) of waste samples after experiment.

The difference above shows that more decomposition of organic material was achieved due to leachate recirculation and optimal moisture content in the waste. The comparison of seven-days respiration index (RI_7_) of all reactors with respect to the limit value for landfill stabilization (completion of aeration) is shown in Figure 12.

For comparison, the results of different parameters analysed for the fresh synthetic waste sample prepared for this research and degraded waste samples collected from all landfill simulation reactors after the experiment are summarized in Table 7.

### 4.4. Carbon Balance

The carbon balance is conducted by analyzing TOC contained in the waste sample before and after the experiment, monitoring the TOC concentration in leachate sampled, and gas flow rate and composition (CH_4_ and CO_2_). All reactors analyzed the initial amount of organic carbon as 413 GC/kg DM. The highest carbon discharge in the liquid phase (leachate) was observed from reactor R2-ACT, where 39 GC/kg DM was mobilized in leachate during the 448 days of anaerobic operation.

The lowest value of carbon discharge in leachate was noted in reactor R4-MOD with a value of 16 GC/kg DM, followed by R3-MOD, where 19 GC/kg DM carbon was mobilized in the liquid phase. In reactor R1-ACT, 21 GC/kg DM carbon mobilized in leachate, the second-highest mobility of carbon in the liquid phase. The highest carbon gasification was observed in R1-ACT with an 88 GC/kg DM value, followed by R3-MOD with a 75 GC/kg DM carbon discharge rate. Both reactors were aerated after the anaerobic phase.

**Figure 13:**
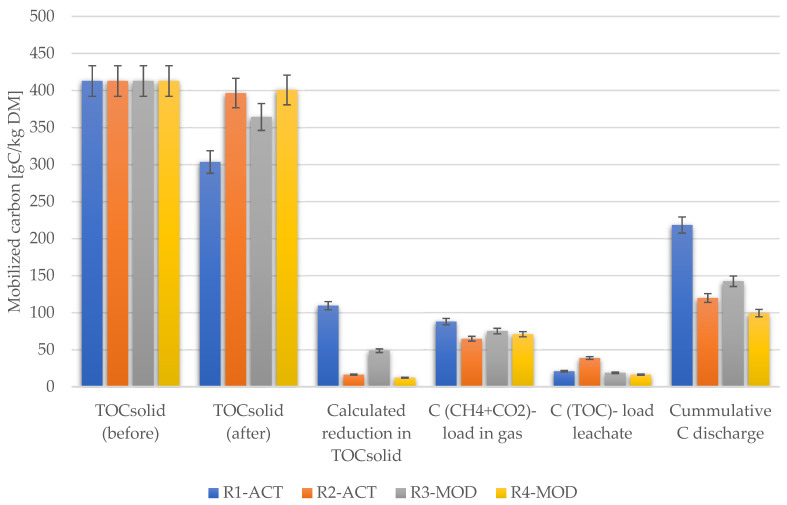
Carbon mobilization during LSR operation.

The lowest level of carbon discharge through the gas phase was recorded from R2-ACT operated under anaerobic conditions throughout the experiment, where 65 GC/kg DM was mobilized with biogas. In reactor R4-MOD, the total quantity of carbon gasification was determined as 71 GC/kg DM. Figure 13 shows the carbon discharge through liquid and gas phases during the pre-aeration, anaerobic, and post-aeration operations conducted during the experiment in respective landfill simulation reactors. A study [76] reported similar observations regarding carbon discharge, where the lowest carbon gasification occurred in the reactor column operated under continuous anaerobic conditions during the test, and the highest carbon gasification was observed in the aerobic column. Moreover, the authors also reported that the anaerobic reactor column showed the highest carbon discharge through leachate as analogously observed in reactor R2-ACT in this study.

The higher carbon reduction in the solid waste was observed in reactors R1-ACT and R3-MOD with total reduction of 109 gC/kg DM and 49 gC/kg DM, respectively, where the post aeration phase was realized. Whereas in reactors operated under anaerobic conditions R2-ACT and R4-MOD, only 16 gC/kg DM and 12 gC/kg DM carbon was reduced. According to Ritzkowski and Stegmann [81], carbon conversion rate is significantly influenced by the ecosystem surrounding microorganisms (including oxygen concentration, pH, temperature, and moisture content) and presence of biodegradable organic matter in waste mass.

The highest cumulative carbon reduction was noticed from reactor R1-ACT, where cumulatively 53% of TOC was reduced from the initial quantity. Second, reactor R3-MOD showed higher carbon mobilization with 35% total carbon discharge from the initial amount. In contrast to this, the total carbon reduction in R2-ACT and R4-MOD was noted as 29% and 24%, respectively, from the initial amount of carbon loaded in reactors. Similarly, study [58] observed higher TOC reduction (31.2%) in solid waste from aerated LSR than from anaerobic LSR (21.5%).

In the overall comparison, the cumulative carbon discharge was higher in aerated landfill simulation reactors than anaerobic reactors due to fact that the metabolism rate in aerobic conditions is significantly higher than in anaerobic conditions [82], resulting in an enhancement in the rate of carbon conversion and stabilization of organic content [60]. The carbon balance and mobilization during landfill simulation reactor (LSR) operation is graphically illustrated in Figure 13.

## 5. Conclusions

Based on the results obtained from landfill simulation reactor R2-ACT simulating the open dumpsite conditions in the situation (annual rainfall rate and MSW composition) in Karachi, it is estimated that solid waste disposed of at dumpsites has the potential to produced landfill gas of approximately 187 m^3^/tonne DM (dry mass) with average methane concentration of up to 57.1% (*v*/*v*). Furthermore, through analysis of these results and MSW disposal situation in Karachi (amount and moisture content), it is estimated that the quantity of MSW disposal annually at dumpsites in Karachi is contributing about 3.9 million tonnes CO_2_-eq. methane emissions (with specific methane potential of 1.8 tCO_2_-eq./tonne DM disposed).

Furthermore, the results of gas formation potential (GP_21_) and respiration activity (RI_4_) investigations of the fresh waste samples showed that the MSW directly disposed of at dumpsites in Karachi is above the recommended stabilization levels for waste material permitted to final disposal in the landfills. Comparing the GP_21_ and RI_4_ results with recommended German waste stabilization criteria for landfilling, it is discovered that the fresh waste samples produced 92% higher biogas than the suggested limit for GP_21_. Furthermore, the results of the respiration index analysis showed that the fresh waste has about 94% higher respiration activity than the suggested limit. After the experiment, residual gas potential and respiration activity of the waste samples obtained from each reactor were investigated. The gas generation potential and extent of waste stabilization of each landfill approach simulated in this study were compared with suggested criteria and target values of GP_21_ and RI_7_ for the completion of landfill aeration operation. The results showed that sanitary anaerobic landfill conditions with a post aeration phase represented by in R1-ACT reactor have higher residual GHG mitigation and waste stabilization potential. The GP_21_ value noted from waste the material sampled from R1-ACT reactor was 6.5 L_N_/kg DM, which is noticeably lower than target value of the GP_21_. Moreover, the target value for the respiration index RI_7_ was also achieved in the waste with 2.5 mgO_2_/g DM.

Second, bioreactor landfill conditions with post aeration represented by reactor R3-MOD show a higher GHG mitigation potential. The waste sample from the reactor R3-MO achieved a target value GP_21_ of 9.4 L_N_/kg DM. However, the respiration activity value for the waste was higher than the limit value, with RI_7_ value of 4.5 mgO_2_/g DM. The least GHG mitigation potential was noted in bioreactor landfills without post aeration, represented by reactor R4-MOD. The GP_21_ value was above the target limit with 14.8 L_N_/kg DM. The respiration activity in the waste sampled from the reactor R4-MOD was also higher than the proposed limit, with RI_7_ value of 4.8 mgO_2_/g DM. Finally, the waste sampled from reactor R2-ACT representing open dump conditions (without active control) showed high GP_21_ and RI_7_ as 19.1 L_N_/kg DM and 5.3 mgO_2_/g DM, respectively, significantly over the proposed model criteria. Based on the results obtained from this study, it is concluded that the reactor simulating the sanitary landfill with post aeration showed higher mitigation potential of residual GHG emissions and waste stabilization. In addition, the LSR with post aeration followed this trend. However, bioreactors with the post-aeration approach showed high and speedy gas production during the anaerobic phase, and higher waste stabilization was achieved due to post-aeration.

To improve the solid waste management situation and optimize the GHG mitigation potential of landfills, this study recommends employing an integrated solid waste management approach in Karachi with comprehensive financial, legal, administrative, and institutional support. Further pilot and field-scale studies must be conducted in the future to optimize GHG mitigation potential of the bioreactor and sanitary landfills with the post-aeration option in Karachi.

## Figures and Tables

**Table 1:** Composition of synthetic MSW sample used in landfill simulation experiment [31].

Waste Component	FW	GW	Paper	Glass	Metal	Plastic	Fines	Nappies	Textile	TP	Wood
Fraction in sample [% *w*/*w*]	26.1	17	8	5.6	1.1	8	3.7	9.8	7.6	10	3.1

**Table 2:** Physicochemical characteristics of modelled MSW sample—adapted from [57]; adapted with permission from Ref. [59]. Copyright 2021 Elsevier B.V.

Sample	Total Solids—TS	Volatile Solids—VS	Total Carbon—TC	Total Organic Carbon—TOC	Total Kjeldahl Nitrogen—TKN	Particle Size
	(% Fresh mass)	(% Dry mass, DM)	(% DM)	(% DM)	(mg/g DM)	(mm)
Fresh MSW	44.4	82.8	41.42	41.28	20.8	≤25

**Table 3:** Basic details of LSR loading with synthetic waste sample—adapted with permission from Ref. [59]. Copyright 2021 Elsevier B.V.

LSR#	Waste Mass (Wet)	Waste Mass (Dry)	Waste Volume	Density (Wet)	Density (Dry)
	[kg]	[kg]	[L]	[kg/L]	[kg/L]
**R1-ACT**	2.80	1.24	4.52	0.62	0.28
**R2-ACT**	3.00	1.33	4.16	0.72	0.32
**R3-MOD**	3.40	1.51	4.65	0.73	0.32
**R4-MOD**	2.80	1.24	4.26	0.66	0.29
**Average**	3.00	1.33	4.40	0.68	0.30

**Table 4:** **Operation** summary of pre-aeration phase (adapted from [57]).

LSR#	Duration [Days]	Aeration Rate [L/kg DM/d]	Water Recirculation	Leachate Analysis	Water Exchange	Off Gas Analysis
All	16	145	Twice per day	Once a week	No	Twice per week

**Table 5:** Summary of the anaerobic and aerobic (post-aeration) operation of LSR experiment adapted with permission from Ref. [59]. Copyright 2021 Elsevier B.V.; adapted from [65].

Operation	Unit	R1-ACT	R2-ACT	R3-MOD	R4-MOD
Anaerobic (An-A)	[days]	252	448	252	364
Post-aeration (Post-AE)	[days]	196	-	112	-
Total operation time	[days]	448	448	364	364
Average water addition	[mL/week]	56	56	165	165
Leachate recirculation	[daily]	-	-	Twice	Twice
Aeration rate (Post-AE)	[L/kg DM/day]	8.28	-	6.87	-

**Table 6:** Estimation of greenhouse gas emission potential of waste disposed of at dumpsites in Karachi.

Parameter	Value	Unit	Reference
Results of lab-scale experiment			
Landfill gas production	187	m^3^/tonne DM	Present study
Methane fraction in LFG	57.1	%	Present study
Methane production	106.8	m^3^/tonne DM	Present study
Amount of MSW annually disposed			
Total MSW generation	15,600	tonnes/day	[24,33]
Moisture content	55	%	Present study
Waste disposal rate	70	%	[45]
MSW disposal	10,920		
Waste dry mass disposal	6006	tonnes/day	
	2,192,190	tonnes/year	
	2.2	Million-tonnes/year	
GHG emissions from MSW annually disposed			
Methane quantity	234,075,471.6	m^3^/year	
	234.1	Million-m^3^/year	
Density of methane	0.66	kg/m^3^	
	154,489,811.3	kg	
	154,489.8	tonnes	
	0.15	Million-tonnes	
GWP of methane	25	CO_2_-eq	[79]
Specific GHG emission	1.8	tonnes CO_2_-eq	
Total GHG emissions	3.9	Mt CO_2_-eq	

**Table 7:** Summary of solid waste characteristics before and after LSR experiment.

Parameter	Unit	Fresh Sample	R1-ACT	R2-ACT	R3-MOD	R4-MOD
Waste mass—initial	[kg]		2.8	3	3.4	2.8
Waste mass—final	[kg]		1.06	1.88	2.8	2.4
TS	[%]	44.42	33.24	36.03	26.7	26.04
VS	[%]	82.85	56.74	74.09	62.34	69.54
TOC	[%]	41.28	30.34	39.65	36.42	40.06
GP_21_	[L_N_/kg DM]	251.9	6.47	18.88	9.34	14.35
BMP_21_	[L_N_/kg DM]	170.1	3.45	9.87	4.36	7.01
Degree of Decomposition	[%]	0	72.1	48.5	61	37.5
RI_4_	[mgO_2_/g DM]	81.8	2.02	3.04	3.06	3.40
RI_7_	[mgO_2_/g DM]	116.7	2.55	5.30	4.54	4.87
Calorific value	[J/g]	19,120	13,508	16,951	16,268	17,937

## Data Availability

Not applicable.
